# Supplementary material for: COZOID: contact zone identifier for visual analysis of protein-protein interactions
Source: BMC Bioinformatics. 2018 Apr 6;19:125. doi: 10.1186/s12859-018-2113-6 (PMC5889581; doi:10.1186/s12859-018-2113-6)
Supplement: Supplementary file 2 — Software build. Executable binary file of the software tool. (ZIP 118,784 kb) [file 12859_2018_2113_MOESM2_ESM.zip › cozoid/platform/docs/testng-6.5.1-javadoc.zip!/org/testng/TestListenerAdapter.html]

TestListenerAdapter (TestNG)


---


|  |  |  |
| --- | --- | --- |
| **Overview** \| **Package** \| **Class** \| **Tree** \| **Deprecated** \| **Index** \| **Help** | |  |
| **PREV CLASS**   **NEXT CLASS** | **FRAMES**    **NO FRAMES**     **All Classes** |  |
| SUMMARY: NESTED \| FIELD \| CONSTR \| METHOD | DETAIL: FIELD \| CONSTR \| METHOD |  |


---


## org.testng Class TestListenerAdapter

```
java.lang.Object
  org.testng.TestListenerAdapter
```

**All Implemented Interfaces:**: IConfigurationListener, IConfigurationListener2, org.testng.internal.IResultListener, org.testng.internal.IResultListener2, ITestListener, ITestNGListener

---

``` public class TestListenerAdapter extends java.lang.Object implements org.testng.internal.IResultListener2 ```

A simple ITestListener adapter that stores all the tests
that were run. You can retrieve these results with the
following methods:
getPassedTests()
getFailedTests()
getSkippedTests()
If you extend this class in order to override any of these
methods, remember to call their super equivalent if you want
this list of tests to be maintained.

**Author:**
:   Cedric Beust, Aug 6, 2004, Alexandru Popescu

---

**Constructor Summary**

`TestListenerAdapter()`


| **Method Summary** | |
| --- | --- |
| `void` | `beforeConfiguration(ITestResult tr)`             Invoked before a configuration method is invoked. |
| `protected  ITestNGMethod[]` | `getAllTestMethods()` |
| `java.util.List<ITestResult>` | `getConfigurationFailures()` |
| `java.util.List<ITestResult>` | `getConfigurationSkips()` |
| `java.util.List<ITestResult>` | `getFailedButWithinSuccessPercentageTests()` |
| `java.util.List<ITestResult>` | `getFailedTests()` |
| `java.util.List<ITestResult>` | `getPassedTests()` |
| `java.util.List<ITestResult>` | `getSkippedTests()` |
| `java.util.List<ITestContext>` | `getTestContexts()` |
| `void` | `onConfigurationFailure(ITestResult itr)`             Invoked whenever a configuration method failed. |
| `void` | `onConfigurationSkip(ITestResult itr)`             Invoked whenever a configuration method was skipped. |
| `void` | `onConfigurationSuccess(ITestResult itr)`             Invoked whenever a configuration method succeeded. |
| `void` | `onFinish(ITestContext testContext)`             Invoked after all the tests have run and all their Configuration methods have been called. |
| `void` | `onStart(ITestContext testContext)`             Invoked after the test class is instantiated and before any configuration method is called. |
| `void` | `onTestFailedButWithinSuccessPercentage(ITestResult tr)`             Invoked each time a method fails but has been annotated with successPercentage and this failure still keeps it within the success percentage requested. |
| `void` | `onTestFailure(ITestResult tr)`             Invoked each time a test fails. |
| `void` | `onTestSkipped(ITestResult tr)`             Invoked each time a test is skipped. |
| `void` | `onTestStart(ITestResult result)`             Invoked each time before a test will be invoked. |
| `void` | `onTestSuccess(ITestResult tr)`             Invoked each time a test succeeds. |
| `void` | `setAllTestMethods(java.util.List<ITestNGMethod> allTestMethods)` |
| `void` | `setFailedButWithinSuccessPercentageTests(java.util.List<ITestResult> failedButWithinSuccessPercentageTests)` |
| `void` | `setFailedTests(java.util.List<ITestResult> failedTests)` |
| `void` | `setPassedTests(java.util.List<ITestResult> passedTests)` |
| `void` | `setSkippedTests(java.util.List<ITestResult> skippedTests)` |
| `java.lang.String` | `toString()` |

**Methods inherited from class java.lang.Object**

`clone, equals, finalize, getClass, hashCode, notify, notifyAll, wait, wait, wait`

**Constructor Detail**

### TestListenerAdapter

```
public TestListenerAdapter()
```


**Method Detail**

### onTestSuccess

```
public void onTestSuccess(ITestResult tr)
```

:   **Description copied from interface: `ITestListener`**
:   Invoked each time a test succeeds.

    :   **Specified by:**: `onTestSuccess` in interface `ITestListener`
    :   **Parameters:**: `tr` - `ITestResult` containing information about the run test **See Also:**: `ITestResult.SUCCESS`

---


### onTestFailure

```
public void onTestFailure(ITestResult tr)
```

:   **Description copied from interface: `ITestListener`**
:   Invoked each time a test fails.

    :   **Specified by:**: `onTestFailure` in interface `ITestListener`
    :   **Parameters:**: `tr` - `ITestResult` containing information about the run test **See Also:**: `ITestResult.FAILURE`

---


### onTestSkipped

```
public void onTestSkipped(ITestResult tr)
```

:   **Description copied from interface: `ITestListener`**
:   Invoked each time a test is skipped.

    :   **Specified by:**: `onTestSkipped` in interface `ITestListener`
    :   **Parameters:**: `tr` - `ITestResult` containing information about the run test **See Also:**: `ITestResult.SKIP`

---


### onTestFailedButWithinSuccessPercentage

```
public void onTestFailedButWithinSuccessPercentage(ITestResult tr)
```

:   **Description copied from interface: `ITestListener`**
:   Invoked each time a method fails but has been annotated with
    successPercentage and this failure still keeps it within the
    success percentage requested.

    :   **Specified by:**: `onTestFailedButWithinSuccessPercentage` in interface `ITestListener`
    :   **Parameters:**: `tr` - `ITestResult` containing information about the run test **See Also:**: `ITestResult.SUCCESS_PERCENTAGE_FAILURE`

---


### getAllTestMethods

```
protected ITestNGMethod[] getAllTestMethods()
```

---


### onStart

```
public void onStart(ITestContext testContext)
```

:   **Description copied from interface: `ITestListener`**
:   Invoked after the test class is instantiated and before
    any configuration method is called.

    :   **Specified by:**: `onStart` in interface `ITestListener`

---


### onFinish

```
public void onFinish(ITestContext testContext)
```

:   **Description copied from interface: `ITestListener`**
:   Invoked after all the tests have run and all their
    Configuration methods have been called.

    :   **Specified by:**: `onFinish` in interface `ITestListener`

---


### getFailedButWithinSuccessPercentageTests

```
public java.util.List<ITestResult> getFailedButWithinSuccessPercentageTests()
```

:   **Returns:**: Returns the failedButWithinSuccessPercentageTests.

---


### getFailedTests

```
public java.util.List<ITestResult> getFailedTests()
```

:   **Returns:**: Returns the failedTests.

---


### getPassedTests

```
public java.util.List<ITestResult> getPassedTests()
```

:   **Returns:**: Returns the passedTests.

---


### getSkippedTests

```
public java.util.List<ITestResult> getSkippedTests()
```

:   **Returns:**: Returns the skippedTests.

---


### setAllTestMethods

```
public void setAllTestMethods(java.util.List<ITestNGMethod> allTestMethods)
```

:   **Parameters:**: `allTestMethods` - The allTestMethods to set.

---


### setFailedButWithinSuccessPercentageTests

```
public void setFailedButWithinSuccessPercentageTests(java.util.List<ITestResult> failedButWithinSuccessPercentageTests)
```

:   **Parameters:**: `failedButWithinSuccessPercentageTests` - The failedButWithinSuccessPercentageTests to set.

---


### setFailedTests

```
public void setFailedTests(java.util.List<ITestResult> failedTests)
```

:   **Parameters:**: `failedTests` - The failedTests to set.

---


### setPassedTests

```
public void setPassedTests(java.util.List<ITestResult> passedTests)
```

:   **Parameters:**: `passedTests` - The passedTests to set.

---


### setSkippedTests

```
public void setSkippedTests(java.util.List<ITestResult> skippedTests)
```

:   **Parameters:**: `skippedTests` - The skippedTests to set.

---


### onTestStart

```
public void onTestStart(ITestResult result)
```

:   **Description copied from interface: `ITestListener`**
:   Invoked each time before a test will be invoked.
    The `ITestResult` is only partially filled with the references to
    class, method, start millis and status.

    :   **Specified by:**: `onTestStart` in interface `ITestListener`
    :   **Parameters:**: `result` - the partially filled `ITestResult` **See Also:**: `ITestResult.STARTED`

---


### getTestContexts

```
public java.util.List<ITestContext> getTestContexts()
```

---


### getConfigurationFailures

```
public java.util.List<ITestResult> getConfigurationFailures()
```

---


### onConfigurationFailure

```
public void onConfigurationFailure(ITestResult itr)
```

:   **Description copied from interface: `IConfigurationListener`**
:   Invoked whenever a configuration method failed.

    :   **Specified by:**: `onConfigurationFailure` in interface `IConfigurationListener`
    :   **See Also:**: `IConfigurationListener.onConfigurationFailure(org.testng.ITestResult)`

---


### getConfigurationSkips

```
public java.util.List<ITestResult> getConfigurationSkips()
```

---


### beforeConfiguration

```
public void beforeConfiguration(ITestResult tr)
```

:   **Description copied from interface: `IConfigurationListener2`**
:   Invoked before a configuration method is invoked.

    :   **Specified by:**: `beforeConfiguration` in interface `IConfigurationListener2`

---


### onConfigurationSkip

```
public void onConfigurationSkip(ITestResult itr)
```

:   **Description copied from interface: `IConfigurationListener`**
:   Invoked whenever a configuration method was skipped.

    :   **Specified by:**: `onConfigurationSkip` in interface `IConfigurationListener`
    :   **See Also:**: `IConfigurationListener.onConfigurationSkip(org.testng.ITestResult)`

---


### onConfigurationSuccess

```
public void onConfigurationSuccess(ITestResult itr)
```

:   **Description copied from interface: `IConfigurationListener`**
:   Invoked whenever a configuration method succeeded.

    :   **Specified by:**: `onConfigurationSuccess` in interface `IConfigurationListener`
    :   **See Also:**: `IConfigurationListener.onConfigurationSuccess(org.testng.ITestResult)`

---


### toString

```
public java.lang.String toString()
```

:   **Overrides:**: `toString` in class `java.lang.Object`


---


|  |  |  |
| --- | --- | --- |
| **Overview** \| **Package** \| **Class** \| **Tree** \| **Deprecated** \| **Index** \| **Help** | |  |
| **PREV CLASS**   **NEXT CLASS** | **FRAMES**    **NO FRAMES**     **All Classes** |  |
| SUMMARY: NESTED \| FIELD \| CONSTR \| METHOD | DETAIL: FIELD \| CONSTR \| METHOD |  |


---
